# Intelligent rehabilitation assistant system to promote the early functional recovery of the elderly patients with femoral neck fracture after hemiarthroplasty (HA): A protocol for a randomized controlled trial

**DOI:** 10.1097/MD.0000000000023078

**Published:** 2020-11-13

**Authors:** Zige Li, Haixiong Lin, Xiaotong Wang, Minling Huang, Junming Feng, Junjie Feng, Junyan Gao, Jingjing Wu, Ziwei Jiang

**Affiliations:** a The First School of Clinical Medicine; b Clinical Medical College of Acupuncture, Moxibustion and Rehabilitation, Guangzhou University of Chinese Medicine; c The First Affiliated Hospital of Guangzhou University of Chinese Medicine, Guangzhou, People's Republic of China.

**Keywords:** electronic information technology, hip fractures, opening and double-dummy RCT, rehabilitation

## Abstract

**Background:**

Femoral neck fracture is a common type of hip fracture, which has a high morbidity and mortality. Surgical treatment is the first choice. However, the functional rehabilitation after operation has not been paid enough attention. In addition, the quality of exercise is difficult to quantify, and the rehabilitation is lack of standards. Therefore, the intelligent rehabilitation assistant system which could record exercise details, might be used to evaluate the quality and adherence to the prescribed exercise to this fragile group of patients has great relevance, so as to provide new ideas for postoperative rehabilitation of hip fracture.

**Methods:**

This is an opening, prospective, double-dummy RCT. Fifty femoral neck fractures patients, older than 65 years and are about to hospitalize for HA, will be invited to study. The sample will be divided into monitoring group and control group randomly at a 1:1 ratio. The prescribed exercises need to be done continuously for 2 weeks. The monitoring group needs additional use intelligent rehabilitation assistant system. Each subject will receive a total of 4 follow-up visits at the designated time (2 weeks, 4 weeks, 12 weeks, and 24 weeks). The following factors will be talked as dependent variables:

1. Hip function, measured by Harris Hip Score (HHS);
2. self care ability, based on the Activity of Daily Living Score (ADL) and The timed “up & go” text;
3. pain, using Visual analog scale (VAS);
4. Postoperative complications and 6-month mortality.

Each subject will receive a total of 4 follow-up visits at the designated time, and the findings will be analyzed statistically considering a 5% significance level (*P* < .05).

**Discussion:**

Exercise under monitor may improve patients compliance and exercise quality, and accelerate the rehabilitation process. This protocol reported in accordance with the CONSORT 2010 checklist and SPIRIT 2013 Checklist.

**Trial registration:**

The trial is registered at Chinese Clinical Trials Registry (ChiCTR2000033213, May 24, 2020).

## 1 Introduction

Femoral neck fractures are subset hip fractures, which accounts for about 3% of whole-body fracture and more than 40% of hip fracture.^[1]^ Hip fractures, considered the most serious results of falls or osteoporosis, usually confirmed in trauma patients with the symptom of hip pain. Every year approximately 1.6 million people over the world sustain hip fractures. In general, the dominant cause of hip fractures is low-energy injuries, and it affected men and women equally.^[2]^ The hip fractures always lead to hospitalization, because of its severity.

A study found that hip fractures take at least 2.35 million disability-adjusted lives each year, and more than 5 million people all over the world experience disability.^[3]^ A displaced femoral neck fracture is a severe injury for all ages. In most cases, it requires surgery. Even if it is not a displaced femoral, the situation is also not optimistic. A systematic review shows that the union rate of patients is 92.6%, the avascular necrosis rate is 7.7% after surgery. For those patients who treated conservatively, the union rate and the avascular necrosis rate is 68.8% and 10.3%.^[4]^ There are many options for the treatment, including internal fixation, hemiarthroplasty (HA), and total hip arthroplasty (THA). HA can help ambulation and function recovery, for the surgeons, it is becoming more likely to be chosen.^[5]^ However, even with the scheduled surgery, the patients still in the risks of subsequent surgical complications, reduced function, hip pain, and reduced health-related quality of life.^[6]^ To reduce the morbidity and mortality caused by hip fracture, not only the treatment for the broken bones but also the treatment for the general weakness is needed.^[7]^

Since the risks of femoral neck fracture increasing exponentially with the age of the patients and the trend of population aging, criteria for treatment measure is needed to ensure the optimal outcomes.^[4]^ The hip fracture patients might be unable to regain independence because of the serious edema of the lower extremity and the reduced knee-extension strength,^[8]^ especially after surgery. Comprehensive rehabilitation could improve mobility, balance, and self-reported activities of daily living. Recently, an increasing number of studies are devoted to lower-limb progressive resistance exercise and balance training to improve several functional outcomes,^[7,9–13]^ such as sitting knee extension and for leg press exercise,^[12]^ strengthening exercises for the hip extensors and abductors, knee extensors,^[10]^ standing- walking practice with eyes closed,^[14]^ etc.

However, as reported, in long-term follow-up studies, conventional rehabilitation programs had no significant effect on falling again or reducing mortality.^[15–17]^ As the matter of fact, with a different understanding of doctors advice, and different health states after a hip fracture, it is inappropriate to confirm the above conclusions from existing analysis under the situation with the ignorance of fulfilling the quality of rehabilitation programs. For example, The Study of Hall WJ^[18]^ arranged 20 minutes -daily training in the morning and evening during the hospitalization for the intervention group, the specific plan included balance, walking exercises, flexion of lower limb joint and individualized progressive resistance. The study showed that although these targeted training could not shorten the length of hospital stay, improve sudden delirium, or reduce the re-entry rate of 90 days, but there was statistically significant benefit soverusual both in the Barthel Index and the SPPB scores.

Therefore, accurately describing the pace of recovery is crucial, which has important use to inform clinical decision-makers and caregivers and patients.^[19]^

In addition, in recent years, more and more researchers have used applications to help guide postoperative rehabilitation of orthopedic diseases, and have achieved good results. For example, Hardt S used an app-based active muscle training program for patients after total knee arthroplasty (TKA) to assess whether the scheduled physical training would improve the outcomes of the immediate postoperative period after lower limb surgery. Throughout their hospital stay, each person of the training group was permanently provided with a prototype of the knee trainer and smart tablet with Genu Sport application for the active knee extension training program, the result shows that this intelligent program could improve the outcome after TKA in the immediate postoperative period, particularly in reducing the pain and improving the range of joint ROM.^[20]^ Although we all acknowledge the above consensus, there are still many difficulties to ensure the patients received an integrated unit with shared care due to the different medical policies and medical forces of various countries.

In this study, the intelligent rehabilitation assistant system was used to record exercise details and assess the quality of rehabilitation. Objective to evaluate the quality and adherence to the prescribed exercise to this fragile group of patients. So that we can continuously monitor the limb movement and better observe the outcome of comprehensive rehabilitation after hip fracture operation. The research hypothesis: regular and quality rehabilitation exercise could improve the limb function and accelerate rehabilitation. This is a new exploration, patient-centered interdisciplinary care of hip fractures, interdisciplinary teamwork involved orthopedic surgery, rehabilitation department, geriatric medicine, anesthesiology, nursing, etc.^[21–23]^

## 2 Methods/design

### 2.1 Study design and setting

**Figure 1:**
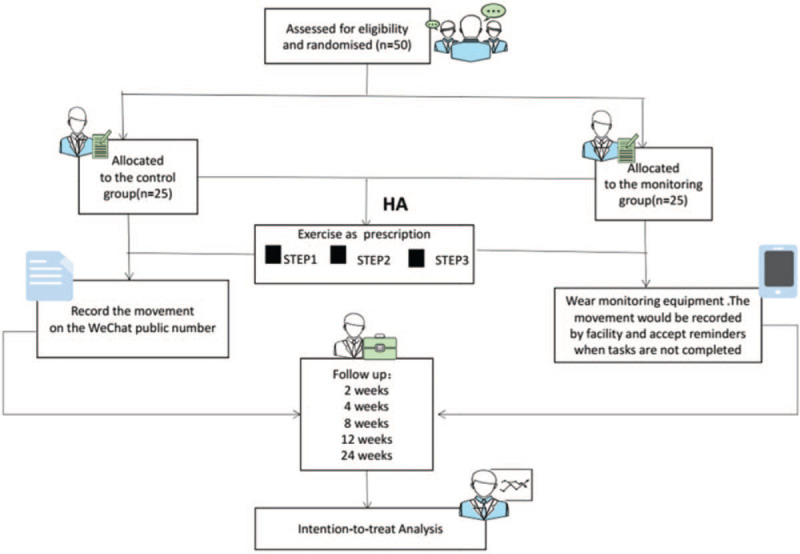
Research flow chart.

This is an opening, prospective, double-dummy RCT. There are 50 patients in total with femoral neck fracture will be recruited from the First Affiliated Hospital of Guangzhou University of TCM, Guangzhou, China. The flow chart and study period are shown respectively in Figure 1. After obtaining written informed consent, the eligible participants will get complete examination before surgery as well as hemiarthroplasty, and then they will be randomly assigned to a monitoring group or a control group in a 1:1 ratio.

### 2.2 Participants

Participants will be recruited via inpatients of a standard-compliant femoral neck fracture, which had a clear history of trauma, hip or greater trochanteric pain, aggravating during activity and was forced to bend the hip and knee, the deformity of pronation and adduction was common. Participants should meet the X-Ray and CT images of a femoral neck fracture. The information about the study, the use of the monitor, and the schedule will be carefully explained before enrollment.

### 2.3 Eligibility criteria

#### 2.3.1 Inclusion criteria

In accordance with the patients with femoral neck fracture were admitted to the hospital from November 2021 to January 2022. The time from this injury to the hospital was no more than 3 weeks.

- Age ≥65.
- The cause of injury is low energy injury.
- After the relevant examination is completed in a hospital, it is judged to be suitable for hemiarthroplasty (the implant materials is a ceramic hip prosthesis, offered by Belang Medical (Shanghai) International Trade Co., Ltd). The operation was performed by the same group of doctors, with more than 5 years working experience, and be able to complete the operation skillfully
- Have certain activity ability before this injury, and can take care of them to some extent.
- According to the X-ray examination, the bone condition of the patients belongs to Singh grade 4 to 6.^[24]^

#### 2.3.2 Exclusion criteria

- Suffered high energy injury or multiple injuries, with multiple fractures in other parts of the body.
- Ipsilateral lower extremity once broken seriously or experienced major surgery.
- Patients with malignant tumors.
- Patients with pathological fractures.
- Patients refused to receive hemiarthroplasty.

### 2.4 Randomization and allocation concealment

The random number will be generated by SAS Institute Inc., Cary, NC, USA, and then put into an opaque envelope, with the attention to concealment of randomization. Participants can be randomly assigned to either group. The participants will receive an opaque envelope given by independent researchers and allocated to one of the 2 groups according to the serial number and group name printed on their treatment card.

### 2.5 Blinding

In this study, patients in the monitoring group need to wear a special motion monitoring devices and interact with doctors online; the implementation of the blind method is difficult, so the form of open clinical research is adopted. However, in the functional test of patients (Harris score of hip joint, life ability assessment, etc.), the evaluators were blinded, and a third person independent of the experiment was invited for data sorting and analysis. The study code will not be revealed until the end of the study unless there is a serious adverse event (AE).

### 2.6 Interventions

**Figure 2:**
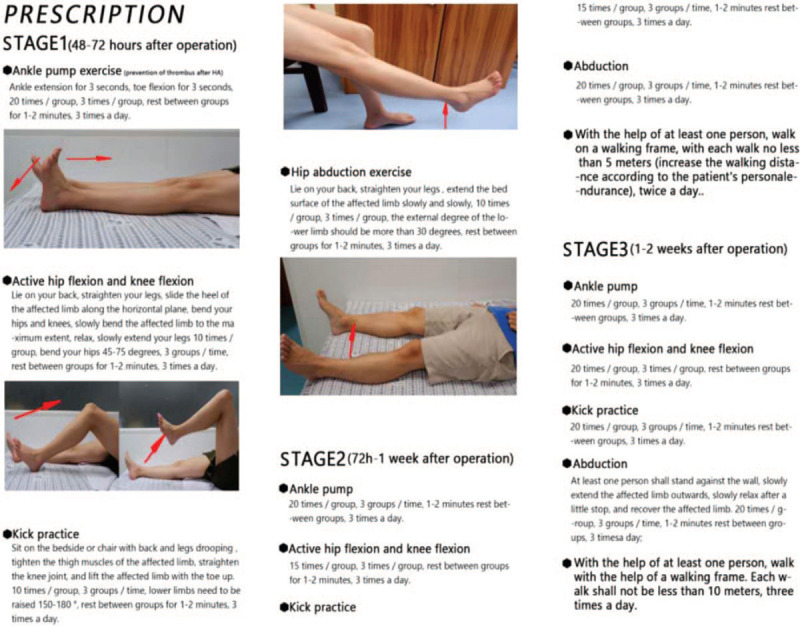
Exercise program.

The participants in both groups will be given the same basic 3-state rehabilitation treatment. Combined with current literature research and clinical experience, the exercise program was developed by 10 senior orthopedic trauma specialists and 1 rehabilitation therapies, which is activity training involves 3 major joints (hip, knee, and ankle), early weight-bearing, gait training and strength training of lower limb muscles. The detailed program will be provided in Figure 2. This exercise prescription is only for the early recovery after HA. On this basis, the amount of exercise can be added as appropriate according to their situation and the doctors advice after 2 weeks. All the subjects received the same routine treatment of prevention of infection, anticoagulation, and pain relief after HA; however, they should not accept a series of exercise programs arranged by other rehabilitation professionals.

#### 2.6.1 The control group

**Figure 3:**
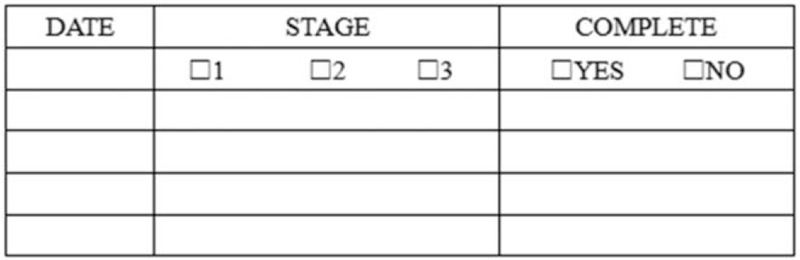
Daily practice record.

The patients of this group of the control group will carry out the staged rehabilitation exercises according to the definite exercise plans, and record the daily exercise in Figure 3 and give it to the doctors during the follow-up. The doctor in charge needs to repeatedly preach and teach during hospitalization to ensure that patients and their families understand the importance of postoperative rehabilitation exercise, and give family exercise guidance after 2 weeks (the same as the experimental group). Regular outpatient follow-up after discharge. If patients have any questions about the rehabilitation process, they should call for a consultation, and do not take the initiative to call for follow-up. Two weeks, 1 month, 3 months, and 6 months after the operation, an outpatient face-to-face examination will be carried out.

#### 2.6.2 The monitoring group

The following interventions were implemented based on the control group: patients should additional use intelligent rehabilitation assistant system (including lower limb motion monitoring devices and interconnect display terminals). The system can collect patients movement times, the amplitude of movement, lower limb weight, and so on, and upload feedback information on the WeChat official account. If patients doubt the rehabilitation process, they could ask the chief physician online questions through the official account. The official account of WeChat will send a reminder of rehabilitation and exercise every day and give the family exercise guidance after 2 weeks (the same as the control group). Two weeks, 1 month, 3 months, and 6 months after the operation, an outpatient face-to-face examination will be carried out.

**Figure 4:**
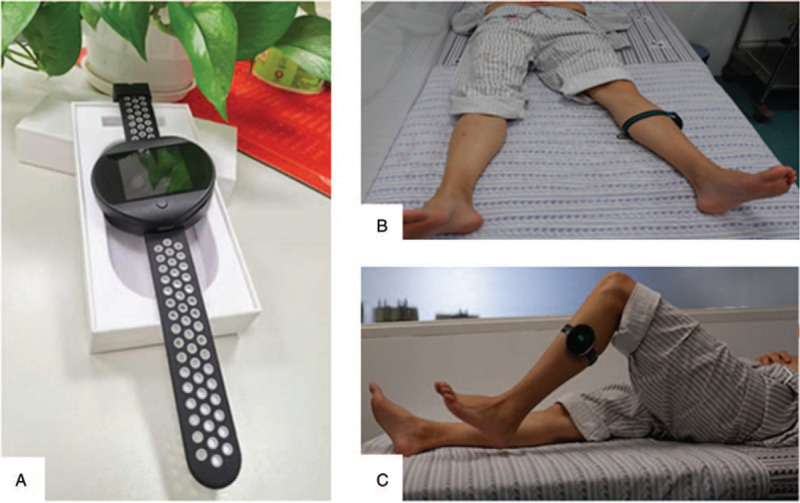
Rehabilitation monitor. Note: a. Physical picture of monitoring equipment, b&c. Exercise with the monitor.

**Figure 5:**
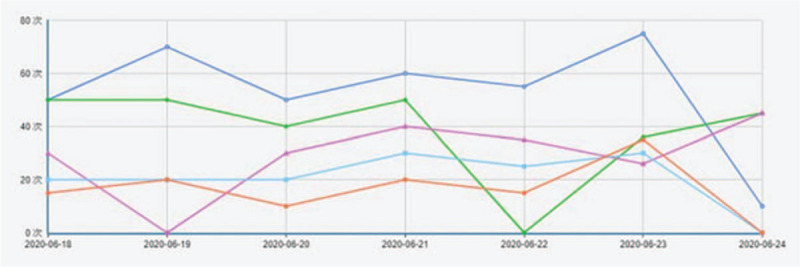
Motion waveform recorded in Wechat background.

The intelligent rehabilitation assistant system involved in this study produced by YILIFE Information Technology Ltd Co, which consists of 2 parts: motion detection equipment and WeChat official account (an interactive platform). The motion detection equipment needs to be worn in different parts of lower limbs, it can observation and exactly record the number of joint movements, range of motion, lower limb muscle strength and walking ability (stride, walking distance), shown in Figure 4 and Figure 5. According to the clinical experience and pre-experimental data, a stander is set up. Only when the practice meets the stander, it could be considered as a successful exercise and recorded as a successful exercise by the machine. For example, you are a patient in stage 1, your ankle extension for 1 second, failed to reach the specified 3 seconds, the monitor will record your motion pattern, but it will not be recorded as a successful exercise. As for the WeChat official account, WeChat is a social software widely used in China, which can be mastered by the elderly. Participants whether in each group would follow the WeChat official account under the guidance of researchers, and researchers will release a video tutorial on rehabilitation exercises. Participants in the control group should record their daily exercise, as for the monitoring group participants, they could get practice feedback from monitor records and be reminded of the task they have completed or not.

### 2.7 Outcome measurement

#### 2.7.1 The primary outcome

The primary outcome is a change in the Harris Hip Score (HHS). HHS is used most commonly as a postoperative functional evaluation tool for hip fractures. The following 7 symptoms can be evaluated by HHS: the degree of the pain, the daily activity function, gait, walking aids, walking distance, deformity degree, and activity scope. The score is judged and recorded by researchers with a totally 100 (excellent: 90∼100, fine: 80∼89, secondary: 70∼79, and not satisfactory: <70), the higher scores indicating better joint function.

#### 2.7.2 The secondary outcome

The Secondary outcome measures are as follows:

- Activity of Daily Living (ADL): it refers to the necessary activities that a person carries out every day to meet the needs of daily life, including eating, dressing, washing, bathing, toileting, dressing, etc. Functional movement includes turning over, sitting up from bed, transferring, walking, driving a wheelchair, going up and downstairs, etc. The total score is 100 (100 points: life self-care; 60 points and above: basic self-care; 40 to 60 points: life needs assistance; 20 to 40 points: life needs great assistance; less than 20 points: life completely dependence.)
- The timed “up & go” text: a fast and quantitative evaluation method of functional walking ability only needs a chair and a stopwatch. During the assessment, the patient is sitting on the chair with the back of the chair and hands on the armrest. If the walking aid is helpful, the walking aid is held in the hand. The reference object set 3 meters away from the seat. With the tester sends out the command of “start”, the patient stands up and walks to the reference point and returns. There is none physical help during the test. The tester records the time taken (in seconds) and the risk of falling during the completion of the test. Before the formal test, the patient is allowed to practice 1 to 2 times to ensure that the patient understands the whole test process. Researchers need to record the complete time of the experiment and evaluate the risk of completing the action. (1 point: normal. 2 points: very slight abnormality. 3 points: slight abnormality. 4 points: moderate abnormality. 5 points: severe abnormality.)
- Visual analog scale (VAS): The subjects participating in the study will indicate the intensity of their pain using a VAS of 100 mm. They must signal on a horizontal line of 100 mm where they would place their pain, where 0 mm indicated “no pain” and 100 mm would be “the worst pain imaginable”.
- Postoperative complications: Such as pulmonary embolism, lower extremity deep vein thrombosis, loosening, and displacement of the prosthesis, periprosthetic fracture, postoperative infection, etc.
- 6-month mortality after HA:

These variables will be measured in the pre-evaluation, first evaluation (week 2), second evaluation (week 4, short-term), third evaluation (week 12, medium-term), and fourth evaluation (week 24, the end of the study). These evaluations will be carried out by an evaluator trained in these procedures, and the data will be stored in an Excel document.

Baseline information of diagnosis and treatment, such as gender, age, comorbidities, imaging, previous self-care ability, injury mode, fracture classification, and potential previous treatment, such as other site fractures, soft tissue injuries, osteoporosis, etc, will be collected from participants medical records.

### 2.8 Participants timeline

A brief Standard Protocol Items: Rehabilitation and evaluation schedule is provided in Table 1.

**Table 1:** Rehabilitation and evaluation schedule.

	Study Period							
	Enrolment	Allocation	Post-allocation (treatment)			Follow-up (evaluation)		
Timepoint	0 week	HA	1 day	1 week	2 weeks	4 weeks	12 weeks	24 weeks
Enrolment:								
Eligibility screen	×							
Informed consent	×							
Clinical Evaluation and Inclusion-Exclusion Criteria	×							
Allocation		×						
Interventions:								
Manual Therapy Protocol			×	×	×			
Therapeutic Exercise Protocol			×	×	×			
Sham Protocol			×	×	×			
Assessments:								
Demographic Data	×							
Neck Disability Index					×	×	×	×
Visual Analog Scale					×	×	×	×
Pressure Pain Threshold					×	×	×	×
Overall Balance Index					×	×	×	×

### 2.9 Sample size calculation

**Figure 6:**
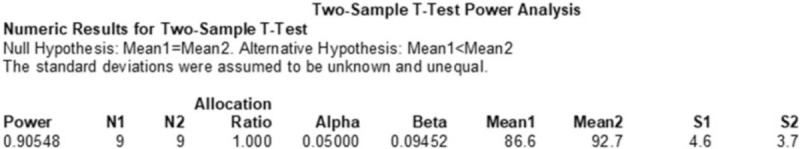
Sample size calculated by PASS 2011.

The sample size is calculated by using the software PASS 2011. Based on the analysis of the variance of means, and estimating an alpha risk of 5% (0.05), a beta risk of 10% (0.10), a unilateral contrast, a typical deviation of 10% (0.10). The primary outcome is the HHS, according to Wang j et al,^[25]^ 6 months after HA, the mean value of Harris score was 86.580 and the standard deviation was 4.629. The experimental assumed the mean value of the monitoring group is 92.650 and the standard deviation is 3.664, and a rate of follow-up losses of 20%, 11 subjects are required in each group, combined with the actual situation of patients in the hospital. Finally, we will include 50 patients divided into 2 groups, each group contains 25 subjects, to overcome this value to assume the possible loss of follow-up, the calculation results are shown in Figure 6.

### 2.10 Data management and statistical analysis

Data are collected from electronic medical records at the study sites, participants study-specific diaries. All collected data for the trial is entered electronically in the encrypted database built by the researchers ourselves which could not be accessed by a third party. The system sets a fixed personal account according to the users organization and role. All attempts to log in to the system will be recorded. All data changes are recorded by user and time, so it can be tracked. All the research data were collected in the First Affiliated Hospital of Guangzhou University of Traditional Chinese Medicine, part of which was recorded in the orthopedic ward and some in the outpatient department. The data management system will check whether all the input data are of correct type, for example, the date must be valid date, and the number must be valid number, at the same time, logical check/verification is designed. Composition of data monitoring committee (DMC) will be undertaken by the Ethics Committee of the First Affiliated Hospital of Guangzhou University of Traditional Chinese Medicine and responsible for reviewing data security once a year.

The statistical analysis will be carried out by IBMSPSS Statistics 21 software. Demographic and baseline data will be summarised using descriptive statistics and graphs as appropriate. Continuous variables will be summarised by descriptive statistics. Categorical variables will be summarised in frequency tables. For the contrast of intragroup hypotheses, Student *t-*test for paired variables will be applied in the case of parametric distribution and Kruskal–Wallis H for nonparametric distributions. Kolmogorov Smirnov test will be used to test the normality of variables. One-factor analysis of variance (ANOVA) will be used in the case of parametric distributions and Kruskal–Wallis H for non-parametric distributions. Statistical tests used to compare between treatments groups will be done two-sided at a signifi-cance level of 5%. The confidence level used will be 95% (0.05), and the power of the study will be 90% (0.1).

### 2.11 Criteria for stopping treatment

Participants could stop the treatment and drop out of the research project for any reason at any time, and the reason for the drop out will be recorded in their CRF. The participants will be told that they have the right to drop out of the experiment and that they will be provided the standardized treatment if they drop out. The criteria for stopping treatment and dropping out from the research project are:

- The participant has suffered from a serious medical disease, and could not receive surgical treatment or rehabilitation exercise according to the plan.
- The participant could not have a regular follow-up or cooperate with telephone follow-up.
- Poor compliance, such as unable to have a regular follow-up or cooperate with telephone follow-up.
- In addition to the standardized treatment of the Department, a whole set of exercise plans of other rehabilitation physicians were implemented.

### 2.12 Ethical approval

The study protocol was approved by the Ethics Committee of the First Affiliated Hospital of Guangzhou University of Traditional Chinese Medicine (NO. ZYYECK [2020] 009), and registered in http://www.chictr.org.cn (ChiCTR2000033213). The study will be explicitly explained to all participants that the experiment involves 2 types of interventions, 2 weeks treatment and 24 weeks follow-up. Be hospitalized to HA is run-in period. All participants will be given sufficient time to decide whether to sign the informed consent form. Written informed consent must be obtained from each participant before they are randomized to a group. If there is any change in the research plan, the study will be suspended and a change application will be submitted to the ethics committee.

## 3 Discussion

Because of the high mortality and disability rate of hip fracture,^[3]^ an increasing number of orthopedic surgeons have realized the importance of available rehabilitation plan of hip fracture after an operation. Compared with internal fixation, HA means earlier loads,^[5]^ it is safer to observe the movement of patients after HA. Although recommend rehabilitation within 48 hours after hip fracture surgery has been widely acknowledged, the current guidelines were unable to answer the following questions: what kinds of exercises are most suitable? How much exercise will be appropriate? And if the patients could accept the prescribed exercise? At present, the published clinical research on rehabilitation exercise after HA, the descriptions of exercise completion quality are less, only a few articles mention about using of load monitoring equipment to measure the weight-bearing strength of the affected limb. In practice, to help patients recover better after HA, orthopedic surgeons can only formulate a Home-Based rehabilitation scheme and use various means to increase the number of follow-up visits and to urge patients to carry out rehabilitation exercises,^[26–29]^ which would burden health care system. The main function of the equipment used in the study is to monitor the completion of the exercise and remind when the exercise is not completed. At the same time, the user's joint movement angle, lower limb weight-bearing force, stride, muscle contraction force, and other contents can be recorded, which provides the basis for our next research.

As far as we know, this is the first clinical study of hip fracture, which is focused on the interaction between early rehabilitation exercise quality and hip function. We designed this opening, double-dummy RCT system, try to obtain a better understanding of the status of rehabilitation exercises for postoperative patients, and observe the relationship between the quality of exercise completion and functional recovery after HA.

## 4 Limitation

In this study, there are also some limitations. First, our study needs the active cooperation of researchers, so it is difficult for us to design a double-blind experiment. Second although the sample size is estimated, monitoring equipment used in our study involving motion monitoring, big data analysis, and communication based on Intern et between doctors and patients, which is an important innovation and quite different from the previous motion monitoring equipment, maybe in the future we have to recalculate the sample size according to the pre experiment results. Third, the current conditions make it difficult to carry out multi center and multi region research. Looking forward the cooperating with more institutions to expand the sample size after the completion of our single center research.

## Author contributions

Conceived and designed the study: Zige Li, Haixiong Lin and Ziwei Jiang. Revised the protocol: Zige Li, Haixiong Lin and Xiaotong Wang. Extracted the data:.Xiaotong Wang. Checked the Data: Zige Li. Performed statistical analysis and wrote the manuscript: Zige Li, Haixiong Lin and Xiaotong Wang. Preparation of clinical study registration materials and draw the charts: Junming Feng, Junjie Feng Interpreted the results: Zige Li, Haixiong Lin, Xiaotong Wang, Ziwei Jiang. Search and organize the literature: Minling Huang, Junyan Gao, Jingjing Wu. All authors contributed constructive comments on the paper.

**Conceptualization:** Haixiong Lin, Ziwei Jiang.

**Data curation:** Li Zige, Xiaotong Wang, Junjie Feng.

**Formal analysis:** Junyan Gao.

**Funding acquisition:** Ziwei Jiang.

**Investigation:** Junjie Feng, Junyan Gao.

**Methodology:** Li Zige.

**Project administration:** Haixiong Lin, Junming Feng.

**Resources:** Minling Huang.

**Software:** Minling Huang, Junming Feng.

**Supervision:** Minling Huang, Jingjing Wu.

**Validation:** Xiaotong Wang, Junyan Gao.

**Visualization:** Xiaotong Wang, Junming Feng, Junjie Feng, Jingjing Wu.

**Writing – original draft:** Li Zige, Junming Feng.

**Writing – review & editing:** Haixiong Lin, Xiaotong Wang, Ziwei Jiang.
